# Arthroscopic Bilateral Autologous Iliac Crest Bone Grafting for Restoring Large Bipolar Bone Loss in Anterior Shoulder Dislocation

**DOI:** 10.1016/j.eats.2025.103652

**Published:** 2025-05-24

**Authors:** Weihong Zhu, Qian Liu, Ding Zhou, Yuchen He, Zhenmu Xu, Shiqi Xiang

**Affiliations:** Department of Orthopaedics, The Second Xiangya Hospital, Central South University, Changsha, Hunan, PR China

## Abstract

In anterior shoulder dislocation, the bipolar bone defect is a severe condition that could impair the stability of the shoulder joint. The bone-grafting technique is an effective method to restore the glenoid defect. For the large defect on the humeral side, several studies have suggested the addition of remplissage procedure; however, there is no consensus. Thus, we describe a bilateral autologous iliac crest bone grafting for patients with severe bipolar bone defect that creates a native anatomical structure and microenvironment for tissue repair. This technique will provide a reliable choice in addressing anterior shoulder dislocation with severe bipolar bone loss.

Because they involve both the glenoid and the humerus, bipolar bone defects represent one of the most challenging types in cases of anterior shoulder dislocation.^1^^,^^2^ Several studies reported that 80% to 90% of patients who undergo arthroscopic examination of shoulder instability notice the bony defect of the glenoid or the humerus.^1^ A bipolar bone defect exacerbates glenohumeral instability, increasing the risk of recurrent dislocation.^3^ Recently, several studies reported favorable results for bone-augmentation procedures (Bristow-Latarjet or autologous iliac crest bone graft) that treat severe types of anterior shoulder dislocation involving a critical glenoid bone defect (>25%) or glenoid bone defect (<25%) combined with critical or off-track Hill-Sachs lesion.^4^^,^^5^ However, the treatment strategy for bony lesions in the humeral head side remains inconclusive. Also, the remplissage technique is one of the options for reinforcing the joint stability.^6^, ^7^, ^8^, ^9^ As a nonanatomical procedure, during the remplissage process, a suture anchor is placed into a Hill-Sachs lesion and tied to the infraspinatus tendon to fill the defect area,^6^ which also invokes concerns, such as the potential loss of range of motion, failing to address the underlying humeral defect, joint stiffness, and longer recovery period before returning to sports.^10^^,^^11^

Here, we propose that using an autologous iliac bone block to reconstruct both glenoid and humerus bone defects to regain the native anatomy structure (Video 1) facilitates the restoration of optimal joint function and improves clinical outcomes for patients with severe bipolar bone lesions.

## Surgical Technique

### Step 1: Preoperative Design

**Fig 1:**
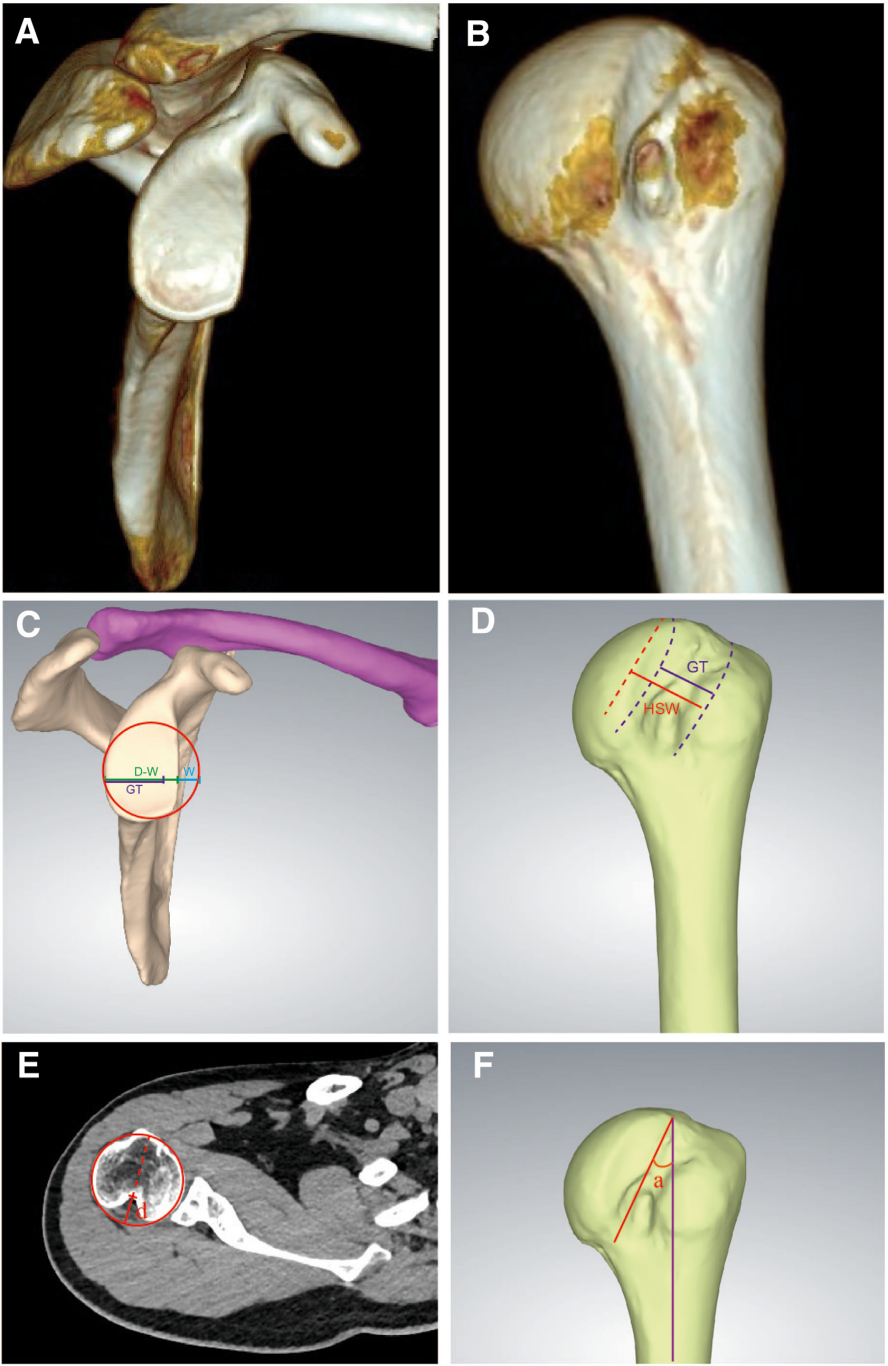
Preoperative computed tomography (CT) scans and 3-dimensional (3D) reconstruction. Shown is the en-face view of right shoulder joint, the glenoid (A), and the posterior view of the humeral head (B) on 3D CT. Computerized 3D construction for the glenoid (C) and humeral head (D). “D” is the diameter of the best-fit circle and the defect width (W) are measured, and “D-W” represents the width of glenoid. The glenoid defect is approximately 23.7%, and therefore the Hill-Sachs bony lesion is defined as “off-track.” The glenoid track (GT) is calculated as 0.83 D-W. “HSW” indicates the width of the Hill-Sachs lesion. (E) On the axial CT image of the humeral head, the diameter of the best-fit circle of the articular surface is measured. In addition, the Hill-Sachs lesion is quantified at its maximal dimension (d). The depth percentage of the Hill-Sachs lesion is found to be 30.9%. (F) The Hill-Sachs angle (a) is defined as the angle formed between the axis of the deepest sulcus of the lesion and the longitudinal axis of the humeral shift.

**Fig 2:**
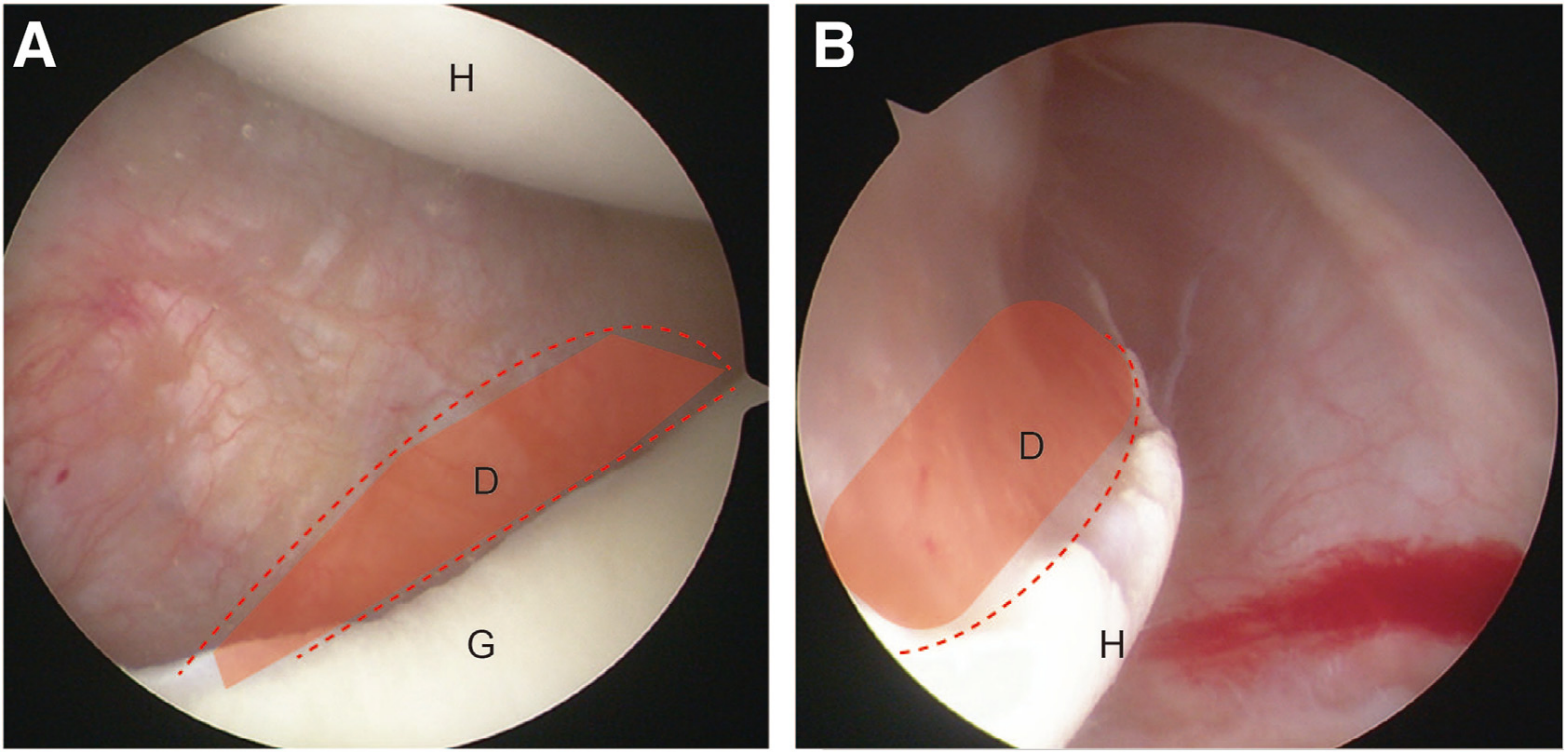
Arthroscopic inspection for the bipolar defect. The right shoulder is shown. (A) The glenoid defect is viewed under arthroscopy through anterosuperior portals. (B) The bone defect on the humeral side is examined under arthroscope from the posterior portal. The red dotted line represents the edge of the bone defect area. (D, defect area; G, glenoid; H, humeral head.)

Before the operation, radiologic examinations including magnetic resonance imaging, computed tomography, and radiographs are performed to thoroughly evaluate the bone defect position, size, and other situations. In addition, 3-dimensional reconstruction and 3-dimensional printing techniques are used for preoperative design as shown in Figures 1 and 2.

### Step 2: Patient Positioning and Arthroscopic Inspection

**Fig 3:**
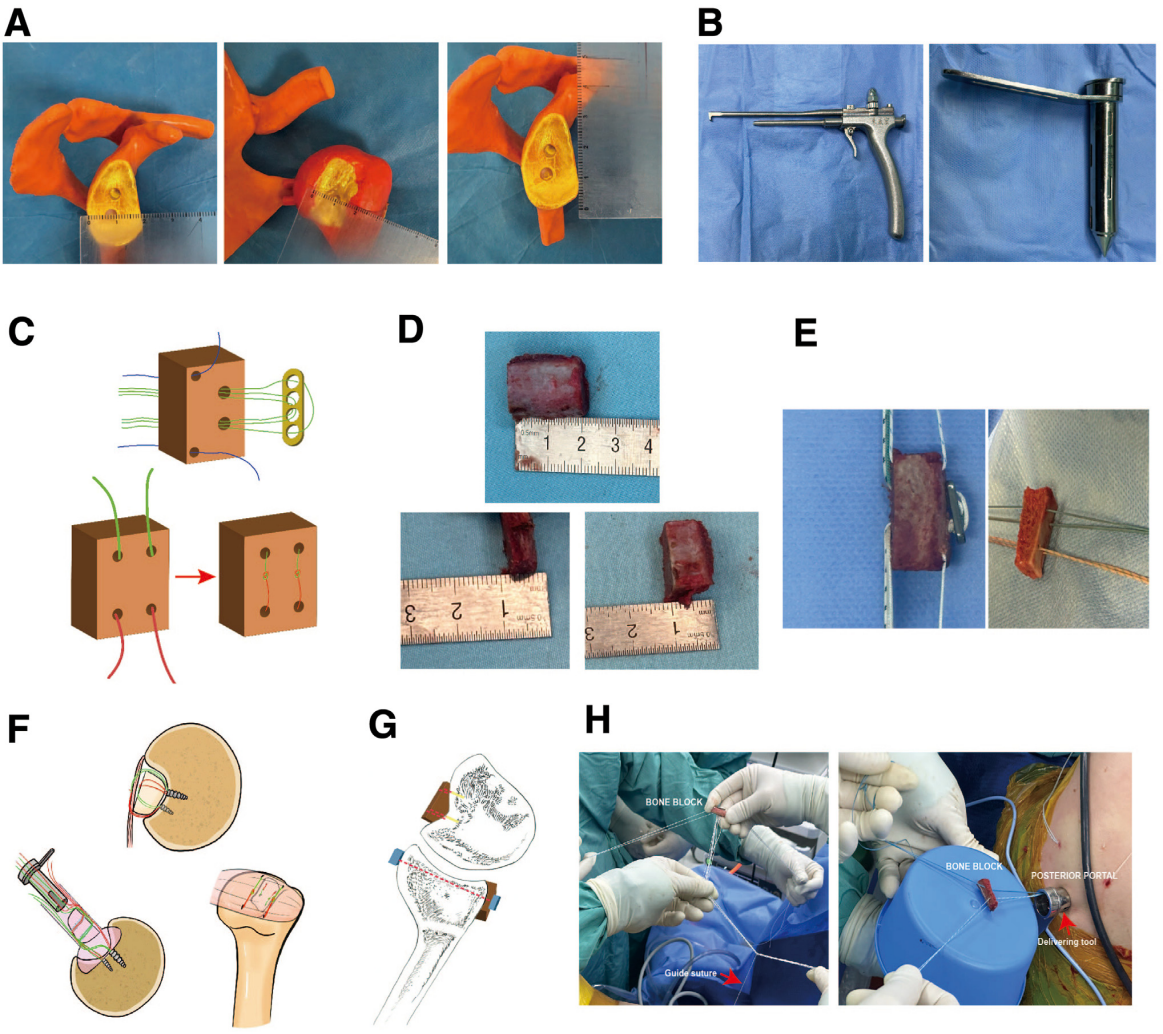
Schematic image for illustration of the bone block grafting. (A) A 3-dimensional printing technique is used for preoperative measurement. The right shoulder is shown. (B) The customized guide tool for drilling glenoid tunnel and delivering the bone block. (C) Three sutures go across the Endobutton plate and the block through the two middle holes. Two individual antirotation sutures go through the other 2 holes. The bone block for the humeral head defect. Four square-shaped tunnels are prepared for 2 sutures. The knots are placed on the bone block to ensure stabilization after grafting. (D) The bone block harvested from the iliac crest. (E) Both bone blocks following suture passage. (F) The schematic procedure of bone block installation for the humeral-side defect. (G) Illustration of the placement of bone graft. (H) The bone block is docked into the tunnel aperture directing to the glenoid defect guided by a suture. The other bone block is prepared for docking onto the humeral defect area through the posterior portal which is created by a customized delivering tool.

The patient is placed in the lateral decubitus position, and general anesthesia is performed. Then, the arthroscope is inserted into the joint through posterior, anteroinferior, and anterosuperior portals, which are established with the cannulas. All conditions of the injury (size, position, inflammatory condition, and location of glenoid defect and Hill-Sachs bony lesion) are examined directly under the arthroscope (Fig. 2). After the confirmation of the requirements for autologous bone graft, a the bone block is prepared (Fig. 3D, E).

### Step 3. Graft Preparation

An incision approximately 3 cm in length is made along the iliac crest above the anterior superior iliac spine. After exposure for the edge of the iliac crest bone, a 2.2-cm × 2-cm × 1.5-cm bone block is harvested by osteotome or oscillating saw. Then, oscillating saw is used to divide the bone block into 2 different pieces according to the size of glenoid defect and Hill-Sachs bony lesion (the size of glenoid bone block is 2.2 cm × 2 cm × 1 cm, the size of humeral bone block is 2.2 cm × 2 cm × 0.5 cm). For reconstruction of the defect on the glenoid, 4 individual tunnels arranged in a trapezoidal pattern are drilled by 1.5-mm and 2-mm Kirschner wires (two 2.0-mm holes is drilled in the middle part of the glenoid bone block, spaced approximately 10 mm; two 1.5-mm holes is drilled at the proximal and distal ends of the glenoid bone block, serving as anchors for anti-rotation sutures). These tunnels are prepared for subsequent fixation using the Endobutton suture fixation system (Smith & Nephew, Andover, MA) and antirotating sutures (Fig 3C). In addition, 4 square-shaped tunnels are drilled into the humeral bone graft in preparation for its placement at the humeral head defect (Fig 3D, E).

### Step 4. Bone Grafting for Glenoid-Side Defect

**Fig 4:**
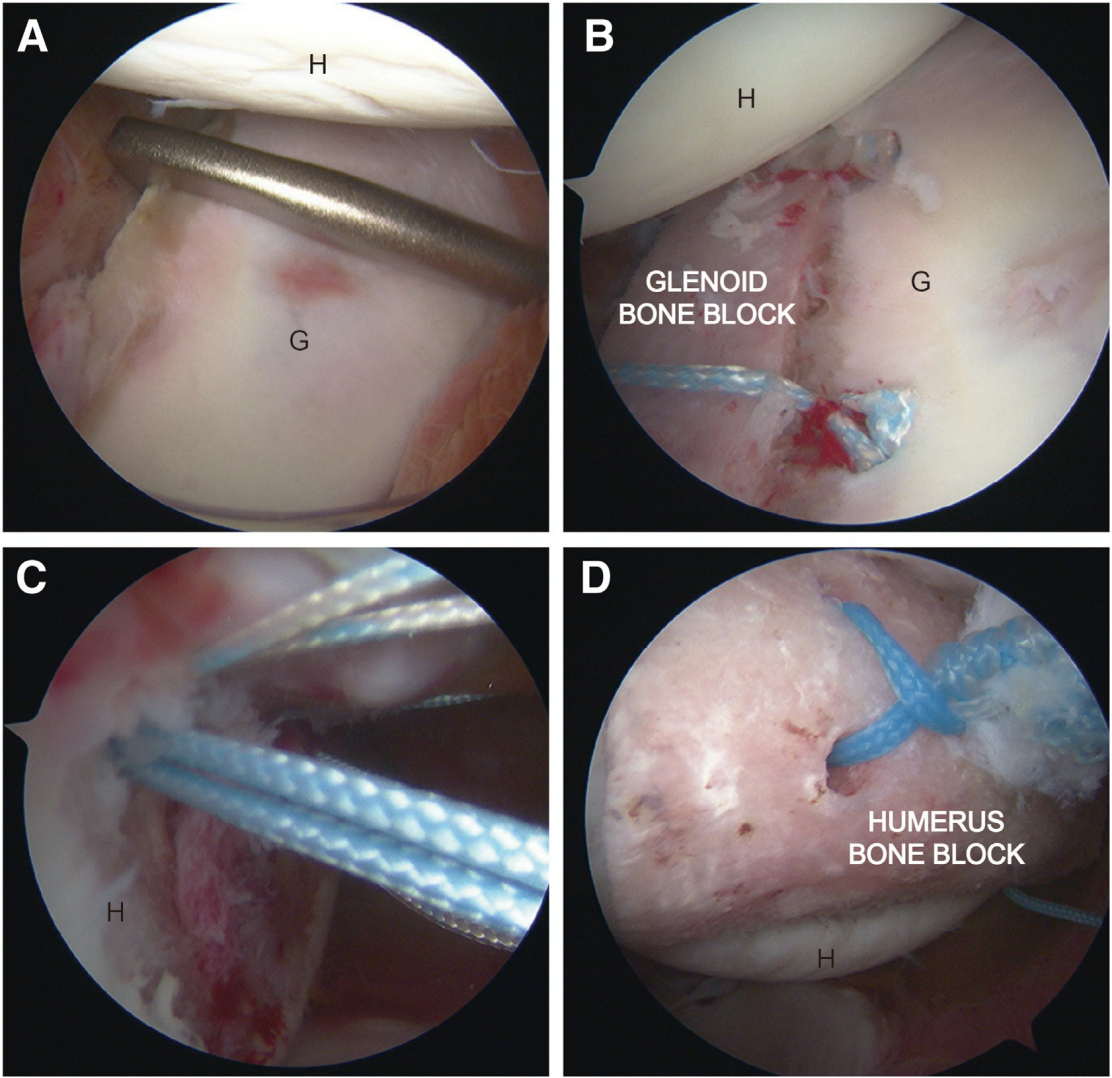
Placement of the glenoid/ humeral graft. The right shoulder is shown. Arthroscopic view from anterosuperior portal: (A) Predocking measurement; (B) post-docking image. Arthroscopic view from the posterior portal: (C) The suture anchors are inserted into the defect area of the humeral head. (D) The placement of bone block on the humeral-side defect. (G, glenoid; H, humeral head.)

The arthroscope is inserted into the anterosuperior portals. After meticulous debridement of the anterior glenoid edge, a customized glenoid tunnel guide tool (Fig 3B) is positioned within the glenohumeral joint. The guide's tip stabilizes the anterior glenoid rim while ensuring the appropriate offset adjustment (Fig 4A). After confirming the proper positioning, a 2.0-mm Kirschner wire is advanced through the guide system to generate a tunnel from the posterior to the anterior aspect of the glenoid. Subsequently, a 4.5-mm Endobutton reamer is introduced along the Kirschner wire to complete the tunnel preparation. A No. 2 high-strength suture is then passed through the glenoid tunnel using a polydioxanone suture after the formation of the 4.5-mm bone tunnel. Once the traction suture is in place, the anteroinferior portals are expanded, and a customized 11-mm metal cannula is used to facilitate the smooth passage of the bone block (Fig. 3H). Three preloaded sutures are shuttled through the bone tunnel from the anterior to the posterior to ensure the correct position of the bone block. Two PushLock suture anchors (2.9 mm; Arthrex, Naples, FL) are placed at the edge of the anterior glenoid with 2 antirotating sutures, respectively, to control the rotation of the glenoid bone block. The switching stick is routinely employed to assist in accurately positioning the bone graft. Once the ideal placement of the bone graft is confirmed, another Endobutton is installed on the posterior aspect of the glenoid (Fig 4B). Typically, sutures from the suture anchors are used to stabilize the anterior capsule at the anterior rim of the glenoid. If necessary, a third PushLock suture anchor is placed between the 2 existing anchors to secure the anterior soft tissue.

### Step 5. Bone Grafting for Humeral-Side Defect

Two 4.5-mm double-loaded suture anchors (HELICOIL; Smith & Nephew) are placed on the superior and inferior margin of central area at the humeral-side defect after the arthroscopic evaluation. Two ends of blue suture at the superior suture anchor are grasped out of the glenohumeral joint space from different capsular portion, 2 ends of different color suture at the inferior suture anchor are grasped with same fashion (Fig 4C). After the glenoid bone block is fixed successfully, the posterior portal is expanded to introduce a customized metal cannula to allow the humeral bone block to pass easily.

The rest of the sutures of the anchors are grasped from the metal cannula. Before the bone graft is transmitted to the humeral defect area, 2 ends of the sutures of the superior anchor are sequentially passed through the superior holes of the humeral bone graft, and the other 2 ends of the sutures of the inferior anchor are passed through the inferior holes (Fig. 3H). The humeral bone block is inserted into the metal cannula with a grasper or clamp. During the passage process, carefully control the direction of the bone block to prevent suture entanglement. The position of the bone graft is adjusted to ensure proper docking. Once the ideal position is confirmed, sutures are tied in a double-pulley fashion to securely attach the bone graft to the humeral defect (Fig 3, Fig 4F, G and 4D). A probe is inserted to assess the stability of the bone graft. Carefully irrigate the joint cavity and close the wound.

## Discussion

**Fig 5:**
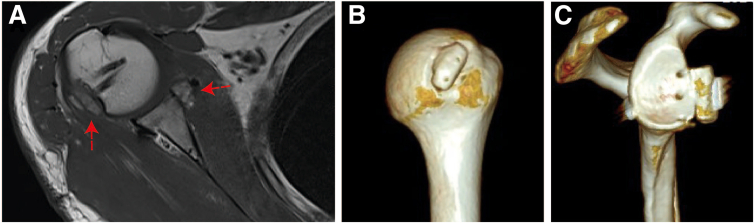
Postoperative computed tomography reconstruction and magnetic resonance imaging. The right shoulder is shown. (A) Cross-section of the shoulder on magnetic resonance imaging after surgery showing the defect filled with bone block. (B-C) Three-dimensional reconstruction of the shoulder after surgery, determined on the basis of the computed tomography scan, showing the graft.

Regarding the bipolar bony defect in patients with anterior shoulder dislocations, numerous studies have been notified the potential cause of it for high recurrence of simple Bankart repair.^12^, ^13^, ^14^ Bone-augmentation surgery has been proven effective for restoring the anatomy of the glenoid and decreasing the recurrent rate.^15^, ^16^, ^17^ In addition, the remplissage procedure has been widely addressed to reinforce the stability of the shoulder joint in case of a huge or off-track humeral head defect. However, in this case, 2 large bone loss were found in both the glenoid (>20%) and humeral head (>25%), and the patient is a fireman who requires high-performance physical activity after surgery, so, in order to minimize the possible impair of postoperative range of motion from this “filling” technique, we describe a bilateral bone grafting technique to restore the anatomical structure rather than directly tighten the soft tissue to the defect area (Figs. 3F, G and 5). Moreover, the double-suture button system and elastic fixations are considered an ideal method for bone block fixation,^18^ so they were also applied for bone block fixation in this technique.

The main features of this technique are as follows: (1) The bone-grafting procedures are undertaken through the arthroscope. (2) Elastic fixation and antirotation anchors provide a stable fixation for both bone blocks on the glenoid and humeral head. (3) As a result of the careful preoperative measurement and the utilization of the delivery instrument, the bone block could be precisely installed on the bone defect area. (4) Compared with remplissage “filling” reinforcement surgery, bony augmentation provides an anatomical for soft-tissue recovery.

The indications and contraindications are shown in Table 1. Pearls/pitfalls are presented in Table 2. The advantages and disadvantages/limitations of our technique are shown in Table 3.

**Table 1:** Indications and Contraindications

Indications
Bipolar bone loss of the glenoid (>20%) and humeral head (>25% or off-track Hill-Sachs lesion), recurrent shoulder dislocation.
Patients with high-quality demands for contact sports.
Contraindications
1. On-track Hill-Sachs lesion.
2. The size of humeral-size bone defect <25%.

**Table 2:** Pearls and Pitfalls

Pearls
The size of the iliac bone block for transplantation is determined on the basis of the 3D-CT scanning and the 3D printing technique, which provides more accurate preoperative measurement.
The installation of bone blocks is transmitted through a customized guide instrument, which allows a smooth docking process.
Before placing the bone block, the appropriate position marker should be made to facilitate the placement procedure under arthroscope.
Once the bone graft is properly positioned at the site of the humeral defect, the graft, the joint capsule, and infraspinatus tendon can be simultaneously compressed to ensure stability.
Hemostatic forceps or posterior portal enlargement are useful for smoothly passing humeral bone block.
Pitfalls
For the installation of the bone block on the humeral head, 2 double-loaded suture anchors should be positioned at appropriate locations, with careful management of each suture stemmed from the 2 distinct anchors; otherwise, improper suture management may lead to suture entanglement and difficult bone block passage.
When drilling and passing the suture through the donor bone block, ensure that the edge of the hole is at least 2 mm away from the edge of the bone block to prevent the bone block breakage.
Improper orientation of the bone graft can lead to challenges in passage and fixation. Preoperative markers on the bone graft and proper application of the switching stick are crucial for ensuring successful graft positioning and secure fixation.

3D, 3 dimensional; CT, computed tomography.

**Table 3:** Advantages and Disadvantages

Advantages
All procedures are managed through arthroscopy (except harvesting iliac crest bone block), which minimizes the trauma.
Regains the anatomical integrity of both the glenoid and the humerus bone, ensuring favorable surgical outcomes.
Achieves an optimal glenohumeral track and prevents the risk of recurrent dislocation by avoiding engagement with the "off-track" zone are essential for successful joint stability and facilitating the postoperative range of motion.
On both sides of the bony lesion, the use of antirotation anchors for iliac crest graft fixation enhances stability, prevents rotation, and effectively promotes the healing of the iliac bone block.
Disadvantages
Nonunion of the bone graft and trauma around the iliac crest harvesting site.
Skilled surgical experience during the procedure is required.

## Disclosures

All authors (W.-H.Z., Q.L., D.Z., Y-C.H., Z.M.X., S.-Q.X.) declare that they have no known competing financial interests or personal relationships that could have appeared to influence the work reported in this paper.

## Supplementary Data

Video 1 The patient is placed in the lateral decubitus position. Then, the arthroscope is inserted into the glenohumeral joint to identify the extend of bone loss of the glenoid and humeral head, and carefully debride the joint space. This patient has an off-track lesion with large bipolar bone loss. Later, a bone block is harvested by osteotome or oscillating saw from the anterior superior iliac crest and divided into 2 different segments according to the size of the defect. Then, two 4.5-mm double-loaded suture anchors are placed on the superior and inferior margins the central area of the Hill-Sachs lesion after the preparation of bone graft. After carefully abrading the anterior edge of the glenoid, a customized glenoid tunnel guide tool is inserted into the joint space and a 4.5-mm glenoid tunnel is created with an Endobutton drill bit. A No. 2 high-strength suture is introduced into glenoid tunnel by a polydioxanone suture. After the high-strength traction suture placement, the anteroinferior portals are expanded by using a customized 11-mm metal cannula to allow the bone block to pass smoothly. Once the ideal location of the bone graft is confirmed, an additional Endobutton is installed on the posterior side of the glenoid. Subsequently, 2 PushLock suture anchors are placed at the anterior edge of the glenoid with 2 antirotating sutures, respectively, to control the rotation of the bone block. Two ends of a blue suture at the superior suture anchor are grasped out of the glenohumeral joint from different capsular portion for remplissage procedure, and 2 ends of different-colored suture at the inferior suture anchor are grasped in the same fashion. The rest of the sutures are passed through the bone block outside the body. The posterior portal is expanded to introduce a customized metal cannula to allow the humeral bone block passage easily. Once the ideal position is confirmed, sutures are tied in a double-pulley fashion to securely attach the bone graft to the Hill-Sachs lesion. Finally, remplissage procedure is completed in the same fashion. Once both bone blocks are docked at the ideal position, the anterior capsule and soft tissue are sutured to the glenoid edge to strengthen joint stability.
